# HAM/TSP in relatives of HAM/TSP cases and in relatives of asymptomatic HTLV-1 carriers

**DOI:** 10.1186/1742-4690-11-S1-P37

**Published:** 2014-01-07

**Authors:** Carolina Alvarez, Kristien Verdonck, Martín Tipismana, Michael Talledo, Jason Rosado, Daniel Clark, Johan Van Weyenbergh, Anne-Mieke Vandamme, Eduardo Gotuzzo

**Affiliations:** 1 Instituto de Medicina Tropical Alexander von Humboldt, Universidad Peruana Cayetano Heredia, Lima, Peru; 2 Rega Institute for Medical Research, Katholieke Universiteit Leuven, Leuven, Belgium; 3 Institute of Tropical Medicine, Antwerp, Belgium; 4 Facultad de Medicina Alberto Hurtado, Universidad Peruana Cayetano Heredia, Lima, Peru; 5 Laboratorios de Investigación y Desarrollo, Universidad Peruana Cayetano Heredia, Lima, Peru; 6 Gonçalo Moniz Research Center, Oswaldo Cruz Foundation, Salvador-Bahia, Brazil; 7 Instituto de Higiene e Medicina Tropical, Universidade Nova de Lisboa, Lisboa, Portugal

## 

To assess the hypothesis that HTLV-1-associated myelopathy/tropical spastic paraparesis (HAM/TSP) runs in families, we compared the frequency of HAM/TSP among HTLV-1-positive relatives of HAM/TSP patients with the frequency of HAM/TSP among HTLV-1-positive relatives of asymptomatic HTLV-1 carriers. We reviewed available information at the Instituto de Medicina Tropical Alexander von Humboldt in Lima (period 1990-2012). Index cases with HAM/TSP were defined as unrelated, HTLV-1-positive patients with a clinical diagnosis of HAM/TSP for whom HAM/TSP was the motive for HTLV-1 testing and who brought ≥1 relative (blood relatives and/or partners) for HTLV-1 testing. Asymptomatic index cases were defined as unrelated, asymptomatic HTLV-1 carriers who were tested for HTLV-1 as candidate blood donors and brought ≥1 relative for screening. In the family studies of 334 index cases with HAM/TSP, 1124 relatives were tested, 318/1124 (28%) were HTLV-1 positive, and 30/318 (9%) had HAM/TSP. In the family studies of 230 asymptomatic index cases, 544 relatives were tested, 204/544 (38%) were HTLV-1 positive, and 15/204 (7%) had HAM/TSP. We classified the relatives in groups based on sex and age. HAM/TSP frequency increased with age and was higher in women than in men. We found no significant differences in HAM/TSP frequency between relatives of HAM/TSP index cases and relatives of asymptomatic index cases. In total, there were 21 families with 2 HAM/TSP cases, 4 families with 3 HAM/TSP cases, and 1 family with 5 HAM/TSP cases. This analysis approach suggests that HAM/TSP usually affects isolated people, but that in some, particular families, HAM/TSP clusters can occur.

